# ClearSee: a rapid optical clearing reagent for whole-plant fluorescence imaging

**DOI:** 10.1242/dev.127613

**Published:** 2015-12-01

**Authors:** Daisuke Kurihara, Yoko Mizuta, Yoshikatsu Sato, Tetsuya Higashiyama

**Affiliations:** 1 Division of Biological Science, Graduate School of Science, Nagoya University, Furo-cho, Chikusa-ku, Nagoya, Aichi 464-8602, Japan; 2 Higashiyama Live-Holonics Project, ERATO, JST, Furo-cho, Chikusa-ku, Nagoya, Aichi 464-8602, Japan; 3 Institute of Transformative Bio-Molecules (ITbM), Nagoya University, Furo-cho, Chikusa-ku, Nagoya, Aichi 464-8602, Japan

**Keywords:** Clearing, Whole plant, Deep imaging, Confocal microscopy, Two-photon microscopy, *Arabidopsis thaliana*, *Physcomitrella patens*

## Abstract

Imaging techniques for visualizing and analyzing precise morphology and gene expression patterns are essential for understanding biological processes during development in all organisms. With the aid of chemical screening, we developed a clearing method using chemical solutions, termed ClearSee, for deep imaging of morphology and gene expression in plant tissues. ClearSee rapidly diminishes chlorophyll autofluorescence while maintaining fluorescent protein stability. By adjusting the refractive index mismatch, whole-organ and whole-plant imaging can be performed by both confocal and two-photon excitation microscopy in ClearSee-treated samples. Moreover, ClearSee is applicable to multicolor imaging of fluorescent proteins to allow structural analysis of multiple gene expression. Given that ClearSee is compatible with staining by chemical dyes, the technique is useful for deep imaging in conjunction with genetic markers and for plant species not amenable to transgenic approaches. This method is useful for whole imaging for intact morphology and will help to accelerate the discovery of new phenomena in plant biological research.

## Abstract (precis)

**Summary:** The optical clearing reagent ClearSee improves the multicolor imaging of fluorescent proteins and dyes and allows the structural analysis of gene expression patterns in multiple plant tissues.

## INTRODUCTION

To understand how cell patterning changes with gene expression, an important challenge in developmental biology is visualization of three-dimensional (3D) morphology with gene expression in intact tissues at the cellular level. Recent advances in fluorescence imaging using fluorescent proteins (FPs), such as green fluorescent protein (GFP), reveal gene expression at the subcellular level. However, it is difficult to observe such FPs in intact plant tissues because plant tissues contain a variety of autofluorescent compounds (Müller et al., 2013), which results in non-specific background fluorescence. In addition, plant tissues contain various components with different refractive indexes (e.g. air, 1.00; cell wall, 1.42; cytoplasm; 1.36) (Kumar and Silva, 1973; Vogelmann et al., 1996). These refractive index mismatches cause light scattering. In traditional observation methods, mechanical sectioning is required to obtain high-resolution images of deep plant tissues. However, it is difficult to reconstruct a 3D representation of gene expression patterns from mechanical sections because of the laboriousness of serial sectioning and potential difficulty of obtaining sections for desired regions and orientations. Classically, a variety of chemical reagents have been used to improve the transparency of plant tissues. Of these reagents, acidified chloral hydrate is most commonly used to clear plant tissues (Lersten, 1967). Chloral hydrate (as Hoyer's solution) has been used for the preservation of specimens since the late 19th century (Hoyer, 1882), and has a high refractive index (1.428), which allows high penetration of light without scattering for a wide variety of plant tissues (Villani et al., 2013). However, to our knowledge, acidified chloral hydrate has not previously been used in conjunction with GFP.

The combination of staining with a chemical dye and clearing with chloral hydrate yields optical sections of high resolution at a subcellular level (Haseloff, 2003). Optical sectioning enables the generation of a series of *z*-stack images, thereby obtaining images in a desired plane after 3D reconstruction. Bougourd et al. (2000) demonstrated the utility of high-resolution (*z*-stacks were collected with 0.2 µm intervals) confocal imaging of mature *Arabidopsis* embryos by clearing with chloral hydrate after staining the cell contents with Aniline Blue. Truernit et al. (2008) performed high-resolution (*z*-stacks were collected with 0.1-0.2 µm intervals) confocal imaging of the cellular structure in various tissues of *Arabidopsis thaliana* by staining the cell membrane with propidium iodide. As alternative approaches for large-scale tissues, optical sections have been obtained by high-resolution X-ray computed tomography (Stuppy et al., 2003), optical projection tomography (Lee et al., 2006) and magnetic resonance imaging (Metzner et al., 2014). However, these techniques lack subcellular resolution. Some of these techniques can be combined with β-glucuronidase (GUS) staining to visualize gene expression at the cellular level (Lee et al., 2006; Truernit et al., 2008), whereas GUS staining cannot be detected at the subcellular level and prohibits the detection of multiple gene expression by multicolor imaging. Recently, array tomography has been developed for 3D imaging at high subcellular resolution, especially *z*-axis resolution, in animal tissues (Micheva and Smith, 2007). Array tomography incorporates automated ultrathin (50-200 nm) sectioning of resin-embedded samples that preserves the fluorescence of FPs, imaging of these sections, and 3D reconstruction. However, application of this method is limited to relatively small specimens.

Multi-photon excitation microscopy (MPEM) is valuable for deep imaging in intact tissues because the excitation wavelength of multi-photon excitation is in the infra-red region, which shows high penetration of biological samples (Centonze and White, 1998). In animal tissues, deep imaging has been achieved by MPEM at 1.4 mm depth for living mouse brain tissue (Kawakami et al., 2013; Horton et al., 2013). Two-photon excitation microscopy (2PEM) has also been used for deep imaging of plant tissues (Feijó and Moreno, 2004). The longer wavelength excitation (1000 nm) for 2PEM allows deep imaging with decreased autofluorescence. Moreover, 2PEM allows multicolor imaging by simultaneous excitation of multiple FPs with a single wavelength, because of the broad two-photon absorption spectra (Drobizhev et al., 2011; Mizuta et al., 2015). However, it is difficult to achieve whole-plant imaging, even by 2PEM, because the complex geometry of plant tissues leads to light scattering caused by refractive index mismatch.

Recently, various chemical mixtures have been used for clearing mammalian tissue to reduce refractive index mismatch and to remove the colored tissue components (Vogt, 2015; Miyawaki, 2015). Sca*l*e, a urea-based aqueous reagent, renders fixed mouse brain samples transparent while preserving the fluorescence of FPs, because urea promotes the hydration of biological samples (Hama et al., 2011). Sca*l*e allows deep imaging over 1.6 mm depth both by confocal imaging and 2PEM. SeeDB, a sugar-based aqueous reagent, clears fixed mouse embryos and brain samples by adjusting refractive index mismatch within the samples without detergents or denaturation reagents (Ke et al., 2013). Sca*l*e requires 2 weeks for clearing of fixed mouse brain samples, whereas SeeDB can shorten the clearing period to 3 days. Surprisingly, CUBIC, a Sca*l*e-based aqueous reagent, allows whole-body imaging as well as whole-brain imaging in mice (Susaki et al., 2014; Tainaka et al., 2014). By chemical screening, aminoalcohol [N,N,N′,N′-tetrakis(2-hydroxypropyl)ethylenediamine] was found to decolorize body samples by solubilization. These reagents have a high refractive index (1.38-1.39 in Sca*l*e; 1.49 in SeeDB; 1.48-1.49 in CUBIC), thereby rendering high transparency to fixed mouse brain tissues (Hama et al., 2011; Ke et al., 2013; Susaki et al., 2014). As alternative approaches for clearing tissues while preserving the fluorescence of FPs, the CLARITY and PACT-PARS methods use active or passive extraction of lipids from the tissue-hydrogel hybrid (Chung et al., 2013; Tomer et al., 2014; Yang et al., 2014).

In this study, we developed an aqueous chemical reagent, termed ClearSee, that renders fixed plant tissues transparent to allow deep imaging by chemical screening. ClearSee rapidly diminishes chlorophyll autofluorescence while preserving the fluorescence of FPs. Multicolor imaging with ClearSee enables observation of the precise 3D structure and specific gene expression patterns. Moreover, ClearSee is applicable to whole-root and leaf imaging using 2PEM and confocal microscopy. We demonstrate the application of ClearSee treatment to whole-seedling imaging for visualization of phloem patterning.

## RESULTS

### Chemical screening of clearing reagents for plant tissues

**Fig. 1.**
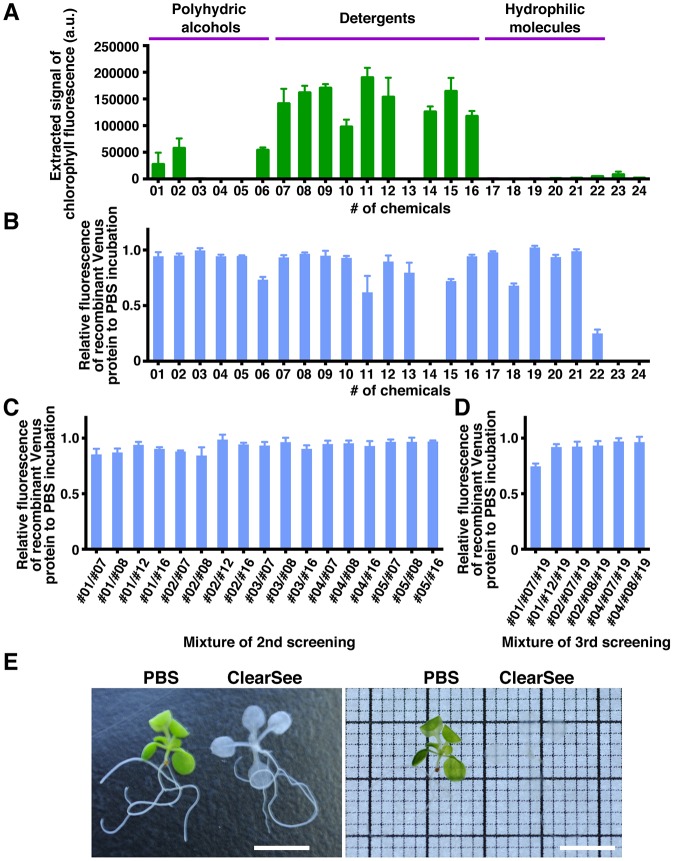
**Screening of chemical clearing solutions for *Arabidopsis* plant tissues.** (A) Fixed leaves were incubated with chemical solutions (#1-24). Autofluorescence of extracted chlorophyll was measured after incubation for 7 days. (B-D) Recombinant Venus proteins were incubated with chemical solutions. The fluorescent signal intensities were measured after 1 day of incubation for the first (B), second (C) and third screening (D). Mean±s.e. shown (*n*=3). (E) Fixed seedlings were incubated in ClearSee or PBS (control) for 2 weeks. In the righthand panel, the samples are shown on the illuminator. Scale bars: 5 mm.

The main source of interruption of fluorescent observation is autofluorescence (e.g. by chlorophyll) in plant tissues (Müller et al., 2013). In the Sca*l*e and CUBIC reagents, polyhydric alcohol/detergent/urea mixtures are used for clearing of brain samples (Hama et al., 2011; Susaki et al., 2014). We first evaluated the clearing efficiency of these compounds for removal of chlorophyll autofluorescence using fixed leaves. We screened 24 compounds, including polyhydric alcohols, detergents, hydrophilic small molecules, and traditional molecules, for clearing (Table S1). We measured the chlorophyll fluorescence at 680 nm emission with 415 nm excitation in the chemical solution over 7 days of incubation using a microplate reader (Fig. 1A). A series of detergents (#07, #08, #09, #11, #12, #14, #15 and #16) showed high activity for chlorophyll extraction with 7 days of incubation. Chloral hydrate (#23) and lactic acid (#24) are among the most commonly used clearing solutions for plant tissues (Simpson, 1929). Because #23 and #24 also quenched chlorophyll fluorescence, these compounds exhibited low activity for chlorophyll extraction in the microplate reader assay (Fig. 1A).

Next, we evaluated the preservative effect of these compounds on recombinant Venus fluorescence (Fig. 1B). In the case of compounds that showed high clearing activity, #14, #23 and #24 strongly quenched Venus fluorescence. Given that some FPs are pH sensitive, we analyzed the FP stability by incubation in neutralized chloral hydrate-based clearing solution with pH adjustment (pH 7.1). The fluorescence of Venus was quenched even with the neutralized chloral hydrate-based clearing solution (Fig. S1). By contrast, the fluorescence of Venus was stable with other compounds, including #07, #08, #09, #12 and #16.

To evaluate the clearing effect of polyhydric alcohols in addition to detergents, we next incubated fixed leaves in detergent/polyhydric alcohol mixtures. For a second screening, #09 was rejected because of the cost. The mixtures of #03/#12, #04/#12 and #05/#12 were not fully mixed, as assessed by visual confirmation, and hence were also rejected because of the low uniformity of the mixtures. The results of the second screening using fixed leaves of UBQ10pro::H2B-mClover are summarized in Table S2. The fluorescence of recombinant Venus was stable in all mixtures (Fig. 1C). Although some #01 mixtures showed high transparency of fixed leaves, the mClover fluorescence was slightly reduced. The #07 and #08 mixtures tended to show high stability of mClover fluorescence and transparency (Table S2). The six combinations that showed high mClover fluorescence, reduced autofluorescence, and high transparency were applied to the third screening. For the third screening, we evaluated detergent/polyhydric alcohol/urea mixtures such as the Sca*l*e/CUBIC reagents (Hama et al., 2011; Susaki et al., 2014). The #01/#07/#19 mixture decreased the fluorescence of recombinant Venus (Fig. 1D). Among the other mixtures, #04/#07/#19 showed high mClover fluorescence, decreased autofluorescence, and high transparency (Table S3). We designated the #04/#07/#19 mixture [10% (w/v) #04, 15% (w/v) #07, 25% (w/v) #19] as ClearSee. Fig. 1E shows a seedling incubated in ClearSee for 2 weeks. Compared with PBS incubation, ClearSee rendered the whole seedling optically transparent.

### ClearSee clears chlorophyll autofluorescence while preserving the fluorescence of FPs

**Fig. 2.**
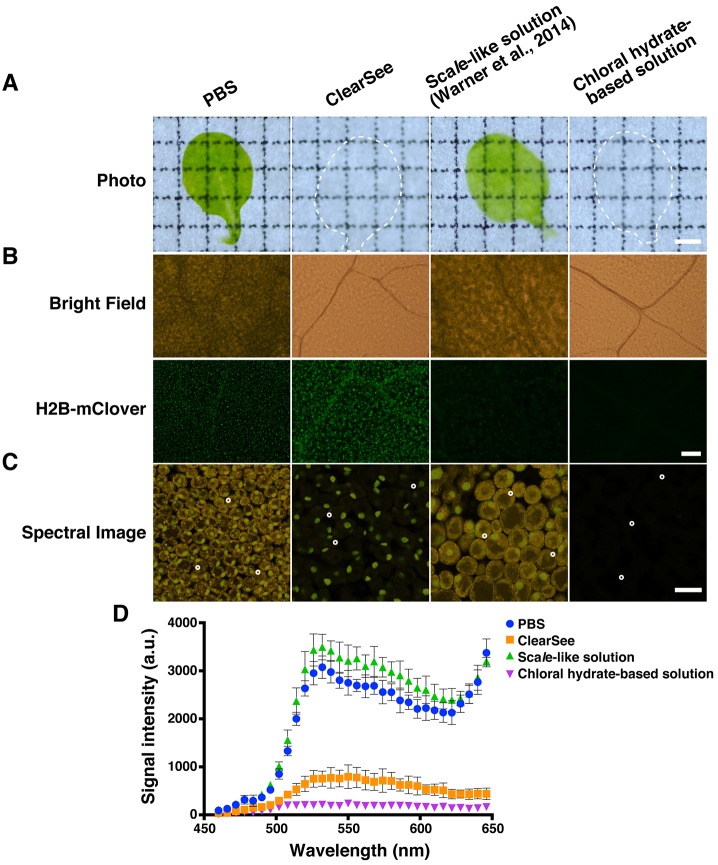
**Optical clearing of *Arabidopsis* leaf using ClearSee.** (A) Fixed *UBQ10pro::H2B-mClover* leaves were incubated in clearing solutions for 4 days and placed on a grid sheet. Note that grid lines are clearly observed in the grid sheet with ClearSee-treated and chloral hydrate-based solution-treated leaf, whereas retention of green coloration and only limited transparency are shown in PBS-treated and Sca*l*e-like solution-treated leaves. (B) Treated *UBQ10pro::H2B-mClover* leaves were observed by fluorescence microscopy. Images of H2B-mClover were acquired using a U-FBNA (excitation 470-495 nm, emission 510-550 nm) filter. (C) Treated *UBQ10pro::H2B-mClover* mesophyll cells were observed by 2PEM with 950 nm excitation. Images were acquired in sequential 6 nm bandwidths spanning the wavelength range 463.9-649.2 nm to generate a lambda stack containing 32 images. (D) Autofluorescence spectrum in leaves treated with various clearing solutions. The measurement regions are indicated by white circles in C. Mean values±s.e. shown (*n*=3 regions). Scale bars: 1 mm in A; 30 µm in B,C.

Recently, Sca*l*e-like solution [6 M urea (#19), 30% (v/v) glycerol (#03), 0.1% (v/v) Triton X-100 (#10)] was used to clear plant tissues (Warner et al., 2014). To evaluate the clearing efficiency of this solution, we incubated fixed leaves in PBS, ClearSee, Sca*l*e-like solution, and neutralized chloral hydrate-based clearing solution. After 4 days of treatment with the clearing solutions, Sca*l*e-like solution-treated leaves still showed green coloration, whereas ClearSee-treated leaves contained no green pigmentation and were transparent (Fig. 2A). The transparency of ClearSee-treated leaves was comparable to that of chloral hydrate-based solution-treated leaves, and indicated that ClearSee rapidly clears leaf tissues.

To examine the stability of FPs with ClearSee treatment, *UBQ10pro::H2B-mClover* leaves were treated with clearing solutions for 4 days. Sca*l*e-like solution did not fully remove the green pigmentation of the leaf, whereas the transparency of ClearSee-treated leaves was comparable to that of chloral hydrate-based solution-treated leaves (Fig. 2B, bright-field). With chloral hydrate-based solution treatment, autofluorescence was dramatically decreased but the fluorescence of H2B-mClover was completely lost (Fig. 2B). By contrast, sufficient fluorescence of H2B-mClover was retained for detection after ClearSee treatment. The nuclei were clearly observed in epidermal cells and vascular bundles (Fig. 2B, H2B-mClover).

To compare the transparency with FP stability for each solution, we performed 3D imaging of *UBQ10pro::H2B-mClover* leaves (Fig. S2). We obtained images from 100 *z*-stacks with 1.0 µm intervals by 2PEM with 950 nm excitation. Although the fluorescence of H2B-mClover was detected to ∼40 µm depth in PBS, the nuclei were clearly observed even at 100 µm depth in ClearSee-treated leaves (Fig. S2A,B). In Sca*l*e-like solution-treated leaves, the signal intensity of H2B-mClover was decreased as depth increased and was difficult to detect at more than 70 µm depth (Fig. S2C). The fluorescence of recombinant Venus was stable in Sca*l*e-like solution (Fig. S1), indicating that the residual autofluorescence of Sca*l*e-like solution-treated leaves prevented the detection of H2B-mClover fluorescence. Consistent with this conclusion, the fluorescence of H2B-mClover was more strongly detected in ClearSee than PBS and Sca*l*e-like solution (Fig. S2A-C). These results indicated that ClearSee renders the leaf tissue transparent while maintaining the fluorescence of mClover.

To examine the causes of autofluorescence, we measured the autofluorescence spectrum with spectral imaging by 2PEM. Autofluorescence was observed in mesophyll cells of clearing solution-treated leaves (Fig. 2C). The two independent emission spectra were detected with 950 nm excitation in mesophyll cells of clearing solution-treated leaves (Fig. 2D). The autofluorescence intensity, especially at >610 nm, was dramatically decreased in mesophyll cells of ClearSee-treated leaves (Fig. 2D). This spectrum corresponded to chlorophyll autofluorescence (Langhans and Meckel, 2014), which indicated that ClearSee diminished chlorophyll autofluorescence while maintaining mClover fluorescence (Fig. 2B,C). The emission peaks in the 500-600 nm range were presumably caused by autofluorescence from the cell wall and other cellular components (Müller et al., 2013; Mizuta et al., 2015). Such autofluorescence was still partly detected in ClearSee-treated leaves (Fig. 2C,D).

### Confocal and two-photon imaging of ClearSee-treated tissues

Recently, we showed that 2PEM is valuable for *in vivo* deep imaging while avoiding autofluorescence in plant tissues (Mizuta et al., 2015). However, 2PEM is not accessible to all researchers because of the equipment cost. To evaluate imaging penetration in ClearSee-treated tissues, we undertook confocal laser scanning microscopy (CLSM) observation of ClearSee-treated roots. Samples were imaged using a 25× water-immersion objective lens [numerical aperture (NA), 1.10; working distance (WD), 2.0 mm]. We obtained images from 150 *z*-stacks with 1.0 µm intervals. Fig. S3 shows optical *xy* and *xz* sections of root tips in *RPS5Apro::tdTomato-LTI6b* lines, in which the plasma membrane is labeled (Mizuta et al., 2015). Although the 2PEM images showed higher contrast than those from CLSM (Fig. S4), both methods were capable of whole-root imaging to almost 100 µm depth (Fig. S3). Fig. S3 shows a comparison of fixed and ClearSee-treated root tips with the same optical setting. Without the ClearSee treatment, the signal intensity was decreased on the opposite side of the epidermis from the objective lens, even in 2PEM images (Fig. S3, fixed). Therefore, ClearSee-treated plant tissues were sufficiently transparent to be penetrated by a single-photon excitation laser (visible laser) and a two-photon excitation laser (Fig. S3).

**Fig. 3.**
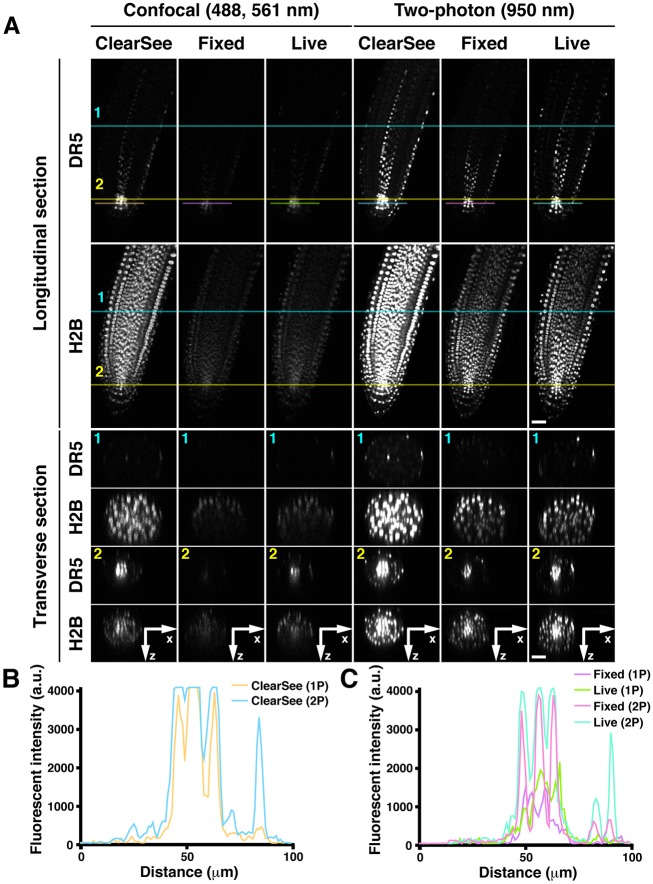
**Comparison of imaging penetration for CLSM and 2PEM in ClearSee-treated *Arabidopsis* root tips.** (A) *DR5rev::3xVenus-N7* (green); *RPS5Apro::H2B-tdTomato* (magenta) root treated with ClearSee for 4 days (ClearSee), or after (fixed) and before (live) fixation without ClearSee treatment. Optical *xy* and *xz* sections were generated from 150 *z*-stack images with 1.0 µm intervals by CLSM with 488 nm and 561 nm excitation (confocal) and 2PEM with 950 nm excitation (two-photon). Beneath are cross-sections at the positions indicated by the colored lines (1, transition zone; 2, meristematic zone). The top of the *xz* section images is facing the objective lens. (B,C) Fluorescence intensities of *DR5rev::3xVenus-N7* recorded at positions 1 and 2. Scale bars: 30 µm.

To determine whether ClearSee allows multicolor imaging and monitoring of hormonal signals, we performed 3D imaging of ClearSee-treated *DR5rev::3xVenus-N7; RPS5Apro::H2B-tdTomato* roots (Fig. 3). The *DR5* promoter marks auxin-responsive transcriptional sites (Ulmasov et al., 1997). As with *RPS5Apro::tdTomato-LTI6b* roots, whole nuclei of the root tip were observed both by CLSM and 2PEM (Fig. 3A, ClearSee). Higher-contrast images of nuclei were obtained by 2PEM than by CLSM, as observed for the plasma membrane (Fig. 3B). The fluorescence of 3×Venus-N7 was observed around the quiescent center in the ClearSee-treated root tip (Fig. 3A, DR5). The expression pattern driven by *DR5rev* in fixed root tips was consistent with that of live root tips (Fig. 3A, fixed, live), indicating that the proper expression pattern was not affected by the clearing processes with paraformaldehyde (PFA) fixation followed by ClearSee treatment. Movie 1 shows reconstructed *xz*-stacks in live and ClearSee-treated *DR5rev::3xVenus-N7; RPS5Apro::H2B-tdTomato* roots by CLSM with 488 nm and 561 nm excitations and by 2PEM with 950 nm excitation. This movie shows that ClearSee allows overall cross-sections of root tips to be obtained optically without sectioning of the specimen. These results demonstrate the advantage of greatly improved transparency achieved by ClearSee treatment for deep imaging and optical sectioning of root tips. We also performed ClearSee treatment for weak expression markers in *Arabidopsis* roots. As shown in Fig. S2, the FPs were more strongly detected in ClearSee-treated samples (Fig. 3A-C). Consistent with this finding, ClearSee-treated *SCMpro::SCM-mGFP5* and *SCRpro::GFP-SCR* roots showed strong GFP fluorescence (Movie 2). These results indicated that ClearSee is also useful for imaging of weak expression markers.

**Fig. 4.**
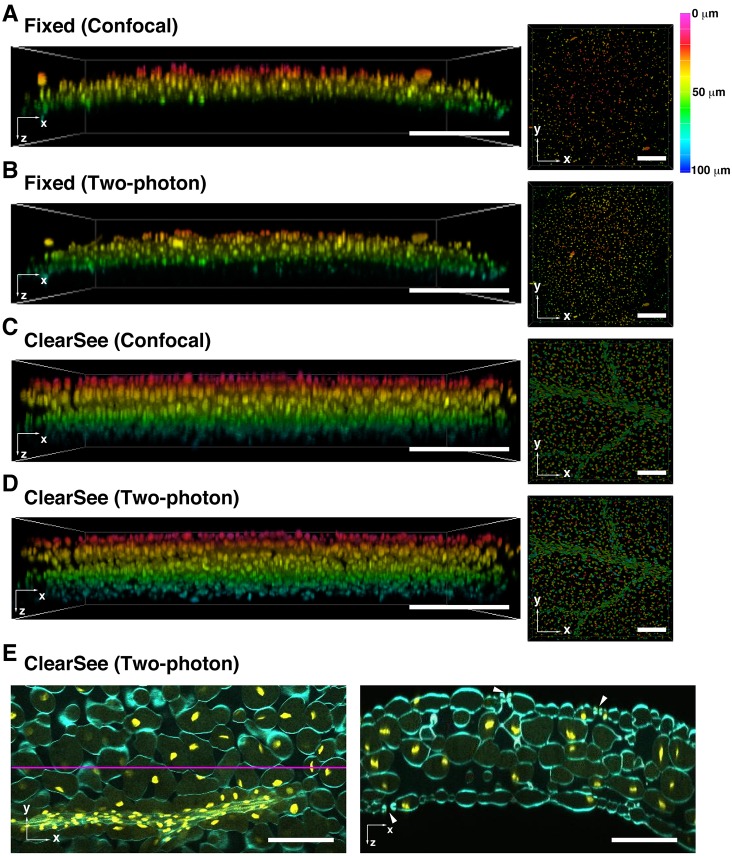
**Application of ClearSee for whole-leaf imaging and staining with chemical dyes.** (A-D) *UBQ10pro::H2B-mClover* leaves fixed with PFA (A,B) and treated with ClearSee for 4 days (C,D). Optical *xy* (left) and *xz* (right) maximum-intensity projections were generated from 100 *z*-stack images with 1.0 µm intervals by CLSM with 488 nm excitation (A,C) and by 2PEM with 950 nm excitation (B,D). The color bar indicates depth from the leaf surface. (E) Cell wall stained with Calcofluor White (cyan) in ClearSee-treated *UBQ10pro::H2B-mClover* (yellow) leaves observed by 2PEM with 950 nm excitation. Left image shows optical *xy* section. The *xz* image on the right is a cross-section at the position indicated by the magenta line. Arrowheads indicate stomata. Scale bars: 100 µm.

Next, we performed whole-leaf imaging. The leaf is a challenging organ for deep imaging because it is composed of multiple cell types, such as epidermal, palisade mesophyll, spongy mesophyll, vascular bundle and guard cells (Littlejohn et al., 2014). As described above, the leaf also contains various components with different refractive indexes, such as cell walls, cytoplasm and air spaces. In addition, different cell types in a leaf exhibit different shapes, orientations, and organelle densities in each cell for efficient light absorption in chloroplasts by internal reflection (Vogelmann, 1986). Therefore, light scattering from these different types of cells and the strong autofluorescence from chloroplasts make deep imaging difficult. As the leaf margins grow, the expression of *DR5::GFP* is detected at the apex of the leaf margin (Bilsborough et al., 2011). Movie 3 shows the ClearSee-treated leaf margin of *DR5rev::3xVenus-N7; RPS5Apro::H2B-tdTomato*. We obtained images from 76 *z*-stacks with 1.0 µm intervals. In the ClearSee-treated leaf, reporter expression under *DR5rev* was clearly detected even at the cellular level in the whole leaf. The fluorescence signals of Venus and tdTomato were detected only in the outer layer of the live leaf margin of *DR5rev::3xVenus-N7; RPS5Apro::H2B-tdTomato*, whereas the expression pattern driven by *DR5rev* in the upper leaf margin was consistent with live and ClearSee-treated leaves (Movie 4). These results suggested that ClearSee preserves specific gene expression, such as that of auxin-responsive genes, at the cellular level in whole tissues. We next obtained images from 100 *z*-stacks with 1.0 µm intervals using the *UBQ10pro::H2B-mClover* leaf. Fig. 4A and B show *xy* and *xz* maximum-intensity projections in the fixed *UBQ10pro::H2B-mClover* leaf without ClearSee treatment. The nuclei were only observed up to 50 µm depth even by 2PEM. Fig. 4C and D show *xy* and *xz* maximum-intensity projections in the ClearSee-treated *UBQ10pro::H2B-mClover* leaf. As shown in Fig. 2A, nuclei were clearly observed in the epidermis and vascular bundles. Although the signal intensity was decreased as depth increased, CLSM detected nuclei in the epidermis on the opposite side from the objective lens to 100 µm depth (Fig. 4C). As observed for root tips, 2PEM showed higher contrast than CLSM (Fig. 4D).

To determine whether ClearSee allows visualization of subcellular components in addition to nuclei and plasma membrane markers, we performed ClearSee treatment of *35Spro::mt-YFP* and *35Spro::GFP-mTalin* leaves, in which the mitochondria and actin cytoskeleton are labeled, respectively (Nelson et al., 2007; Oikawa et al., 2003). The localization patterns of mt-YFP and GFP-mTalin were similar in fixed and ClearSee-treated mesophyll cells (Fig. S5), suggesting that ClearSee is suitable for imaging of subcellular components.

To assess the possibility of post-staining in ClearSee-treated tissues, we stained the cell wall with Calcofluor White in ClearSee-treated leaves. We obtained images from 256 *z*-stacks with 1.0 µm intervals. As shown in Fig. 4E, the cell wall was stained with Calcofluor White even in the mesophyll cells, while maintaining the fluorescence of mClover. The stomatal pores were also observed by *xz* optical cross-section (Fig. 4E, right). In addition, we stained the nuclei with Hoechst 33342 in ClearSee-treated leaves. We obtained images from 144 *z*-stacks with 1.0 µm intervals. As shown in Fig. S6, the nuclei were stained with Hoechst 33342 even in the central mesophyll cells. These results indicated that ClearSee is compatible with staining by chemical dyes.

### Visualization of pistil interior by whole imaging

**Fig. 5.**
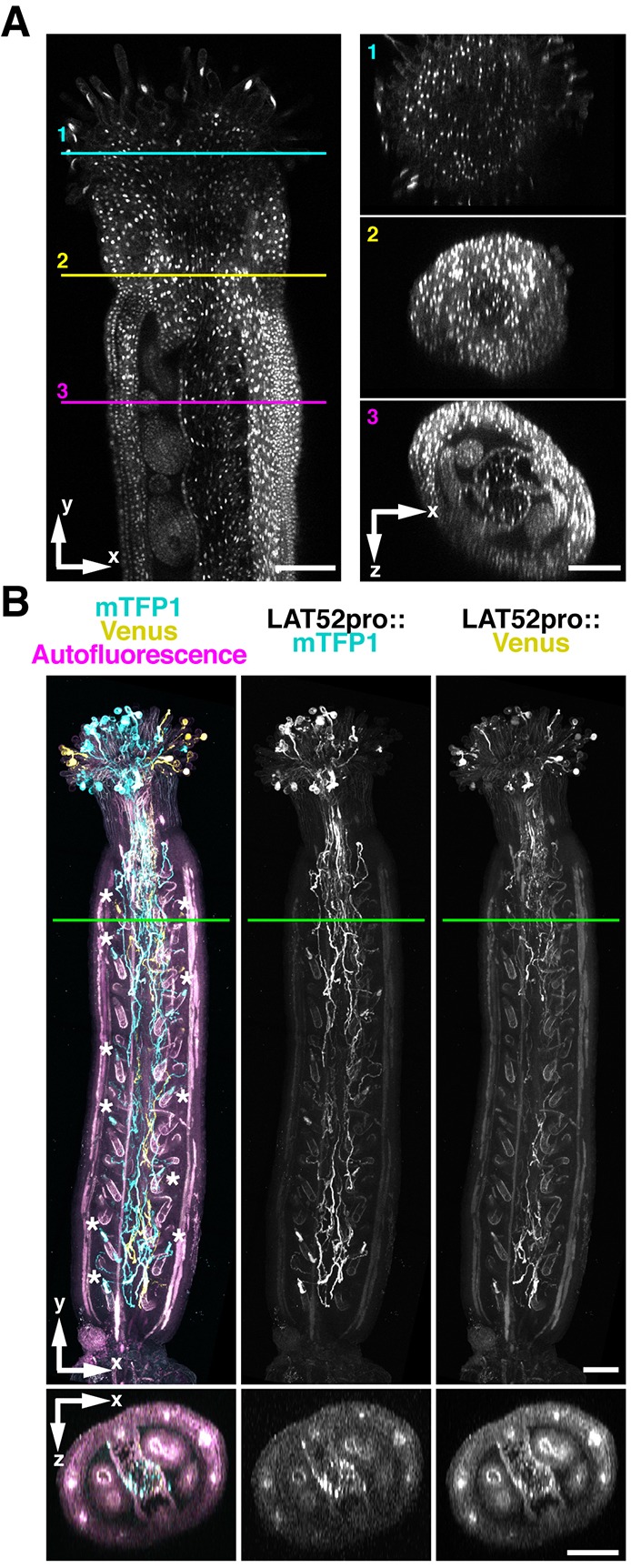
**Application of ClearSee for multicolor imaging of the whole pistil.** (A) *UBQ10pro::H2B-mClover* pistil treated with ClearSee for 6 days. Optical *xy* and *xz* sections were generated from 410 *z*-stack images with 1.0 µm intervals by 2PEM with 950 nm excitation. (B) Pistil pollinated with *LAT52pro::mTFP1* and *LAT52pro::Venus* pollen and treated with ClearSee for 5 weeks. Maximum intensity projections for *xy* view and *xz* sections were generated from 60 *z*-stack images with 6.0 µm intervals by 2PEM with 950 nm excitation. Each image on the left in A and the bottom images in B represent *xz* cross-sections at the positions indicated by the colored lines (1, stigma; 2, style; 3, ovary). Asterisks indicate discharged pollen tubes. The top of the *xz* section images is facing the objective lens. Scale bars: 100 µm.

We next evaluated fluorescence imaging of the ClearSee-treated pistil. Sexual reproduction processes occur in female reproductive organs in the pistil concealed by multiple cell layers, hence it is difficult to observe these important events because of the complex internal structure (Crawford et al., 2007; Cheung et al., 2010). We obtained images from 401 *z*-stacks with 1.0 µm intervals of the fixed *UBQ10pro::H2B-mClover* pistil (Fig. S7A). The nuclei were observed only in the epidermal cells of pistils. We obtained images from 410 *z*-stacks with 1.0 µm intervals in the ClearSee-treated *UBQ10pro::H2B-mClover* pistil (Fig. 5A). The stigmatic papillae are elongated cells with a large nucleus. The style showed a dense structure in spite of penetration by pollen tubes. In the ovary, the transmitting tract showed a sparse structure, caused by programmed cell death (Crawford et al., 2007). The ovules were connected to the margin of the septum. Thus, the precise structure of the pistil was clearly observed after ClearSee treatment without sectioning of the specimen. Movie 5, which shows reconstructed *xz*-stacks in the ClearSee-treated *UBQ10pro::H2B-mClover* pistil, illustrates how ClearSee reveals the complicated internal structure of the pistil and the journey of the pollen tube from the stigmatic papilla to the ovule through the transmitting tract.

**Fig. 6.**
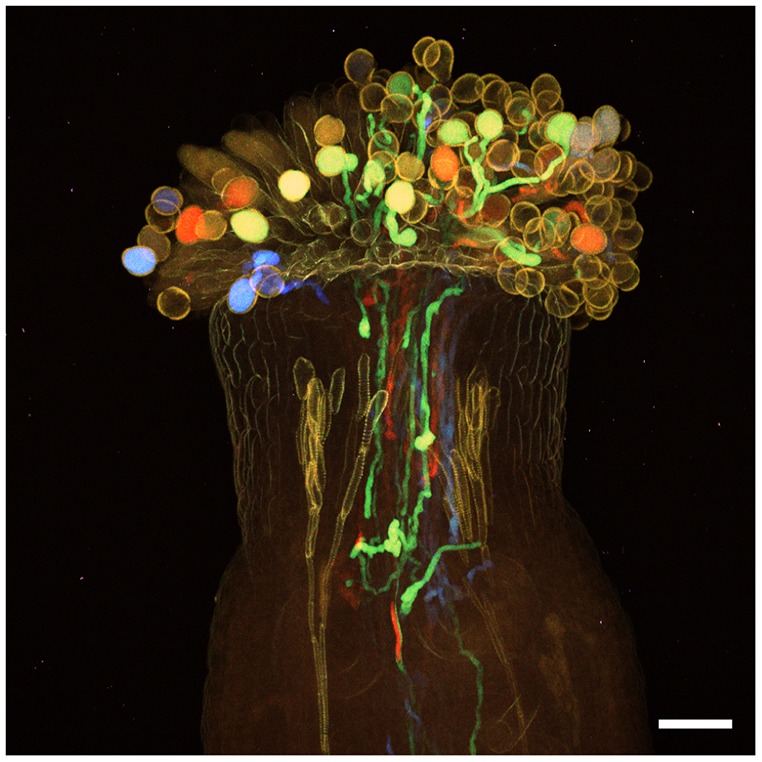
**ClearSee is applicable for long-term storage.** Pistil pollinated with *LAT52pro::mTFP1*, *LAT52pro::sGFP*, *LAT52pro::Venus*, and *LAT52pro::mApple* pollen and treated with ClearSee for 5 months. Maximum intensity projection for *xy* sections was generated from 96 *z*-stack images with 3.0 µm intervals by 2PEM with 990 nm excitation. Images were acquired in sequential bandwidths of 8 nm spanning the wavelength range 460-648 nm to generate a lambda stack containing 19 images. Scale bar: 50 µm.

Next, we performed multicolor imaging of pollen tubes. Previously, the pollen tube has been specifically labeled by staining with Aniline Blue (Cheung et al., 2010), but different genotypes of pollen tubes are indistinguishable with this method. Recently, we performed multicolor imaging by 2PEM using transgenic plants expressing five different FPs, which are simultaneously excited by 2PEM at 980 nm (Mizuta et al., 2015). The pistil was pollinated with pollen from *LAT52pro::mTFP1* and *LAT52pro::Venus*, and then fixed with 4% PFA 6 h after pollination. The pollinated pistil was treated with ClearSee.v2 [v2 differs in that #7 was changed to 5% (w/v)] for 4 weeks. After ClearSee treatment, we obtained images from 60 *z*-stacks with 6.0 µm intervals. Entry of each pollen tube into each ovule was observed within the whole pistil. Discharge of the pollen tube contents was also detected in the ovules (Fig. 5B, asterisks). Pollen tubes expressing mTFP1 and Venus were obviously distinguished, indicating that ClearSee differentiated pollen tubes of distinct genotypes within the pistil. In *xz* optical sections, the position of pollen tubes in the transmitting tract could be observed. In the case of a fixed pistil without ClearSee treatment, the pollen tubes were not detected within the pistil, even by 2PEM (Fig. S7B). Thus, ClearSee is useful for multicolor imaging of different genotypes, ecotypes, and gene expression in deep complex plant tissues. In addition, Fig. 6 shows multicolor pistil imaging after treatment for 5 months with ClearSee.v2. The pistil was pollinated with pollen from *LAT52pro::mTFP1*, *LAT52pro::sGFP*, *LAT52pro::Venus*, and *LAT52pro::mApple*. Spectrum imaging by 2PEM with 990 nm excitation showed that each of the four FPs were clearly distinguishable. It is notable that each pollen tube color was observed even after treatment with ClearSee.v2 for 5 months. This raises the possibility of long-term storage of plant tissues treated with ClearSee.

### Visualization of phloem by whole-seedling imaging

The vascular system extends throughout the entire plant body to supply not only water and nutrients but also signaling molecules (Notaguchi and Okamoto, 2015). Multiscale imaging from the subcellular to the whole-plant is required to assist with understanding the functioning of the vascular system. However, the vasculature is an internal tissue and is therefore difficult to observe with conventional microscopy. The vascular system consists of multiple tissues, such as phloem and xylem (Turner and Sieburth, 2003). Previously, the phloem has been labeled with GUS for staining of whole leaves and seedlings, but GUS-stained images show low resolution at the subcellular level (Bauby et al., 2007). The phloem has also been labeled with *SUC2pro::RCI2A-mCitrine*, which allows high-resolution imaging even at the subcellular level (Thompson and Wolniak, 2008). However, it is difficult to observe the phloem of whole plants by fluorescent imaging, as described above. Therefore, we evaluated whole-seedling imaging for visualization of phloem distribution using *SUC2pro::RCI2A-mCitrine* lines treated with clearing solution.

**Fig. 7.**
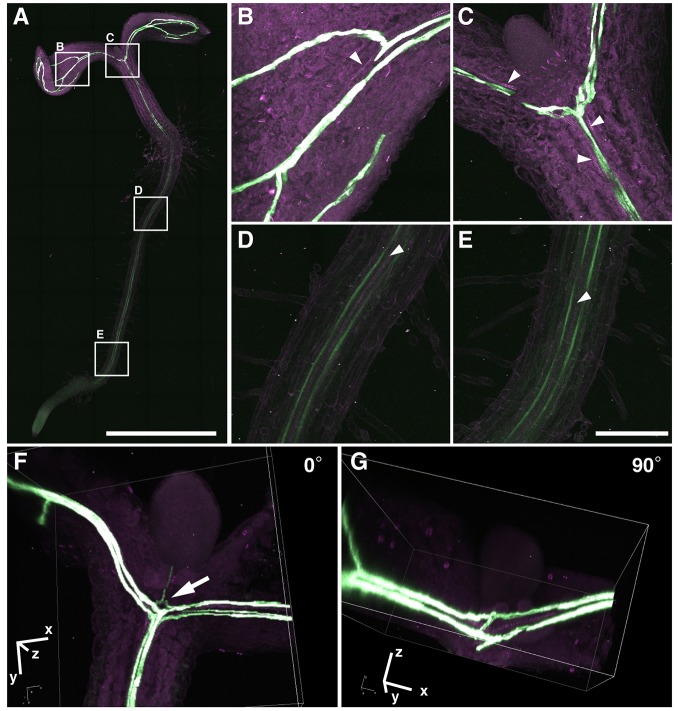
**Phloem patterning in the whole seedling.** (A-E) *SUC2pro::RCI2A-mCitrine* seedling treated with ClearSee for 7 days. Maximum intensity projection for *xy* view was generated from 67 *z*-stack images with 10 µm intervals by 2PEM with 950 nm excitation. Boxed regions in A are magnified in B-E. (F,G) Reconstituted 3D image of seedling with rosette leaves expressing *SUC2pro::RCI2A-mCitrine* after ClearSee treatment for 7 days. Arrowheads indicate spiral xylem vessels. Arrow indicates extension of phloem into rosette leaf from root. Scale bars: 1 mm in A; 100 µm in B-E.

The seedlings with cotyledons were fixed with 4% PFA and then cleared with ClearSee for 7 days. Movie 6 shows *z*-stack images of *SUC2pro::RCI2A-mCitrine* by CLSM. The phloem distribution from the root to the cotyledons was clearly visualized (Movie 6, green), and the spiral secondary wall thickening of xylem vessels was also observed in bright-field images. Fig. 7A-E shows whole-plant images of *SUC2pro::RCI2A-mCitrine* obtained by 2PEM. We obtained the merged image for a 5×10 *xy* tiling array from 67 *z*-stacks with 10 µm intervals using a 25× objective lens. As shown in Fig. 7A, phloem patterning labeled with *SUC2pro::RCI2A-mCitrine* was observed in the whole seedling. Fig. 7B-E shows enlarged images from Fig. 7A, but with the same resolution as Fig. 7A. The phloem was parallel to spiral thickened xylem vessels in the root (Fig. 7B-E, arrowheads). The phloem branched from the root into each cotyledon (Fig. 7C). The venation pattern in the cotyledon was also observed in the ClearSee-treated seedling (Fig. 7B), but not in the fixed seedling (Fig. S8). Fig. 7F,G show ClearSee-treated seedlings of the *SUC2pro::RCI2A-mCitrine* line with rosette leaves*.* Phloem extension into the cotyledons, and subsequently into rosette leaves, was observed (Fig. 7F, arrow). Thus, phloem development patterning was clearly observed after ClearSee treatment. Although clearing takes longer compared with seedlings, ClearSee diminished chlorophyll autofluorescence in adult plants after bolting (Movie 7). Taken together, these results showed that ClearSee is applicable for whole-plant imaging.

### ClearSee is applicable to other plant species

**Fig. 8.**
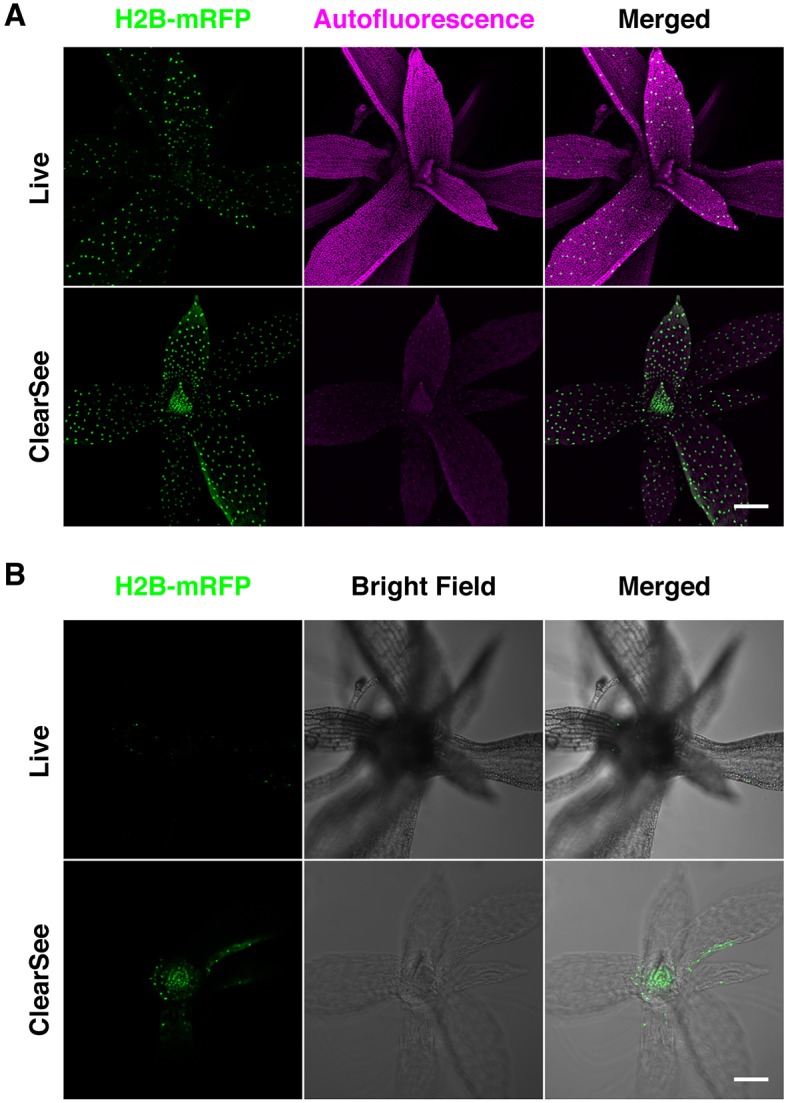
**Clearing of a leafy gametophore of *Physcomitrella patens* with ClearSee.** A leafy gametophore of the H2B-mRFP line of *P. patens* treated with ClearSee for 4 days. Images were collected in the ranges of 570-668 nm for H2B-mRFP and 672-701 nm for autofluorescence with 561 nm excitation by CLSM. (A) Maximum-intensity projections were generated from 325 *z*-stack images with 1.0 µm intervals for living and ClearSee-treated gametophores. (B) Optical slice of the apical region of gametophore covered with juvenile gametophore leaves. Scale bars: 100 µm.

To explore the applicability of ClearSee for other plant species, we cleared the gametophyte of the moss *Physcomitrella patens*. Although the moss protonema, which is the initial stage after spore germination, and the gametophore leaf cells are suitable for cellular and subcellular observation owing to their single-layered structure, observation of the apical region of the gametophore is difficult because of the complicated structure and autofluorescence. Fig. 8 shows the gametophore in the living and ClearSee-treated *H2B-mRFP* line, which was generated by inserting *mRFP* into the *H2B* locus in the wild type. In the living gametophore, strong chlorophyll autofluorescence was observed in the gametophore leaf cells (Fig. 8A, live, autofluorescence). Thus, the structure in the apical region of the gametophore was concealed for both fluorescence and bright-field observations (Fig. 8B, live). By contrast, the intensity of chlorophyll autofluorescence was decreased in the ClearSee-treated gametophore (Fig. 8A, ClearSee, autofluorescence). The H2B-mRFP signal was clearly observed even in the apical region of the gametophore, as well as in the gametophore leaf cells, following ClearSee treatment (Fig. 8B, ClearSee, H2B-mRFP). These results suggest that the ClearSee clearing method is not limited to angiosperm tissues but is also suitable for non-vascular plant tissues while maintaining the stability of FPs.

## DISCUSSION

We developed ClearSee as a clearing reagent for plant tissues to allow deep imaging. Plant tissues are difficult samples for deep imaging because chlorophyll and other cellular contents absorb light, and the complex geometry, including air spaces within tissues such as in the leaf and pistil, diffract light by refractive index mismatch. ClearSee rapidly diminishes chlorophyll autofluorescence and substitutes it with a solution of high refractive index (ClearSee, 1.410; ClearSee.v2, 1.395) in the whole plant body. The method is applicable for a variety of organs, such as the leaf, root, pistil and seedling of *A. thaliana* and the moss *P. patens*. Moreover, ClearSee allows deep imaging of the whole leaf and root, even by CLSM. This finding is advantageous for many researchers because CLSM is more commonly used than 2PEM. In *A. thaliana*, the thickness of the root and leaf is ∼100 µm and ∼150 µm, respectively, and therefore CLSM with ClearSee should be applicable to these depths in other plant tissues. Nevertheless, 2PEM provides higher resolution and signal-to-noise ratio in ClearSee-treated samples, especially for the *z*-axis. Higher *z* resolution is important for 3D reconstruction and optical transverse sectioning. Conventional mechanical sectioning is laborious and time-consuming because fixation and embedding of samples is needed and optimization is tissue and species dependent. In addition, obtaining the desired section planes may be difficult. Fluorescence microscopy with ClearSee enables the suitable *z*-stack images for 3D reconstruction to be obtained, and therefore images of the desired regions and planes with any orientation by optical sectioning. In addition, 2PEM with ClearSee permits deeper imaging, as shown by whole-pistil (∼400 µm) and whole-seedling (∼670 µm) imaging. Deep imaging by 2PEM with ClearSee raises the possibility of whole-plant imaging.

ClearSee diminishes chlorophyll autofluorescence, but the fate of autofluorescence derived from other cellular contents remains unclear. The sources of autofluorescence in the emission range 500-600 nm include phenols, flavins, polyacetylene and isoquinoline in the vacuole, chloroplasts and cell wall (Müller et al., 2013). In mammals, aminoalcohol diminishes the color of heme in the blood by chemical screening (Susaki et al., 2014; Tainaka et al., 2014). This property allows whole-body imaging of mice to be performed with CUBIC, which includes aminoalcohol. Additional screening to identify chemical reagents to clear the residual autofluorescence with ClearSee would permit clearer and deeper imaging in plant tissues.

In the present study, we evaluated the utility of seven FPs (mTFP1, sGFP, mClover, Venus, mCitrine, tdTomato and mApple) and fusion proteins (free FPs, nuclear localization signal, histone and membrane proteins). This versatility of ClearSee could enable the analysis of morphology and cell patterning with multiple gene expression during development. The potential application of ClearSee as a substitute for GUS staining was demonstrated. GUS staining requires optimization of the staining conditions depending on the tissue and the promoter of interest, and staining diffuses from around the exact expression site. In addition, normal DR5 expression was observed with ClearSee, which suggests that hormonal or environmental responses are maintained after ClearSee treatment. Given this applicability of ClearSee, we traced the growth of pollen tubes of different genotypes in the pistil after pollination by labeling with different FPs. Previously, pollen tube guidance within the pistil has been mainly studied using Aniline Blue staining. Aniline Blue clearly stains the pollen tube, but all pollen tubes are stained identically. By contrast, following ClearSee treatment, multicolor imaging can be used to study gene expression in such as the pistil for analysis of cell-cell communications during male-female interactions and between different genotypes and ecotypes.

To date, phloem development has mainly been studied using mechanical sectioning for anatomical observations and/or GUS staining for gene expression (Bonke et al., 2003). However, it is difficult to trace the continuity of vascular strands from mechanical cross-sections and analyze gene expression at the cellular level from whole-mount GUS staining. In ClearSee-treated seedlings, we performed whole-plant imaging to observe 3D structure from micro to macro scales. By obtaining merged images with a 25× objective lens, we could observe the vascular strands throughout the plant body even at the cellular level. Recently, it was suggested that vascular systems have a role in long-distance signaling in response to environmental changes by transferring mobile molecules, such as hormones, peptides and RNA (Notaguchi and Okamoto, 2015). ClearSee will be a useful technique for the study of such long-distance signaling in response to localized changes as it enables whole-plant imaging at the cellular level.

Given the successful clearing of moss tissue by ClearSee, the reagent may be applicable to a wide range of plant species. We also demonstrated that the application of ClearSee is not limited to transgenic plants with FP markers. The applicability of staining with chemical dyes showed that ClearSee could also be used for deep imaging in plant species that are not amenable to transgenic approaches. Moreover, ClearSee is compatible with post-treatment staining with chemical dyes, suggesting that it will permit the incorporation of chemical dyes together with FP markers in transgenic plants. In the case of the pistil, we attempted to use a version with less detergent, ClearSee.v2. Although clearing with ClearSee.v2 required a longer treatment time than with ClearSee, we obtained images with improved clarity. Therefore, the concentration of the individual ClearSee components should be optimized for the specific plant species or tissues under investigation for improved image clarity and depth.

## MATERIALS AND METHODS

### Plant materials and plant growth conditions

For all experiments, *Arabidopsis thaliana* accession Columbia (Col-0) was used as the wild type. The following transgenic lines have been described previously: *RPS5Apro::tdTomato-LTI6b* (Mizuta et al., 2015), *DR5rev::3xVenus-N7; RPS5Apro::H2B-tdTomato* (Heisler et al., 2005; Adachi et al., 2011), *SCMpro::SCM-mGFP5* (CS66496; Kwak and Schiefelbein, 2008), *SCRpro::GFP-SCR* (CS6504; Gallagher et al., 2004), *35Spro::mt-YFP* (mt-yk, CS16264; Nelson et al., 2007), *35Spro::GFP-mTalin* (Oikawa et al., 2003), *LAT52pro::mTFP1*, *LAT52pro::sGFP*, *LAT52pro::Venus*, *LAT52pro::mApple* (Mizuta et al., 2015), *IPT3pro::GFP-GUS* (kindly provided by T. Kakimoto, Osaka University, Japan), and *SUC2pro::RCI2A-mCitrine* (Thompson and Wolniak, 2008).

*A. thaliana* seeds were sown on plates containing half-strength Murashige and Skoog salts (Duchefa Biochemie, Haarlem, The Netherlands), 0.05% MES-KOH (pH 5.8), 1× Gamborg's vitamin solution (Sigma) and 1% agar. The plates were incubated in a growth chamber at 22°C under continuous lighting after cold treatment at 4°C for 2-3 days. Two-week-old seedlings were transferred to soil (Sakata no Tane; Sakata Seed, Yokohama, Japan) and grown at 22°C under continuous lighting.

The H2B-mRFP line of the moss *Physcomitrella patens*, which was generated by inserting *mRFP* into the *H2B* locus in the Gransden 2004 wild-type strain (Rensing et al., 2008), was used. The fragmented protonemata were cultured on BCDAT medium for 4-5 weeks under white light at 25°C and developed into leafy gametophores (Nishiyama et al., 2000).

### Cloning and transgenic plants

For *UBQ10pro::H2B-mClover*, the 634 bp *UBQ10* promoter (upstream of At4g05320), the full-length coding region of *H2B* (At1g07790) fused to mClover (obtained from Addgene plasmid 40259, with A206K mutation introduced) with the (SGGGG)_2_ linker, and the NOS terminator were cloned into the binary vector pPZP211 (Hajdukiewicz et al., 1994). The binary vectors were introduced into *Agrobacterium tumefaciens* strain EHA105. The floral dip or inoculation methods were used for *Agrobacterium*-mediated *Arabidopsis* transformation (Narusaka et al., 2010).

### Chemical screening

First screening was performed using a microplate reader (EnSpire; PerkinElmer) for rosette leaves from *A. thaliana*. Leaves were fixed with 4% (w/v) PFA for 120 min in PBS under vacuum. Fixed leaves were washed in PBS and incubated with 400 µl screening chemical solutions (Table S1) in 96-well plates. After 7 days of incubation, 200 µl were transferred into new 96-well plates and chlorophyll fluorescence measured at 680 nm emission with 415 nm excitation.

The fluorescence stability of Venus in chemical solutions was measured with a microplate reader. To prepare the recombinant Venus protein, the full-length coding region of Venus was cloned into the pCold I expression vector (Takara). The recombinant Venus protein was expressed in *Escherichia coli* strain Rosetta-gami2 (DE3) pLysS (Novagen). After induction with 1 mM isopropyl-β-D-thiogalactopyranoside at 15°C overnight, cells were harvested and lysed in 20 mM phosphate buffer containing 500 mM NaCl, 5 mM imidazole, 1 mM 2-mercaptoethanol, and cOmplete Protease Inhibitor Cocktails (Roche). After sonication and centrifugation, the supernatants were collected. Recombinant Venus was incubated in chemical solutions for 24 h and the fluorescence intensity was measured at 515 nm emission with 485 nm excitation. The refractive index of ClearSee was measured by a digital refractometer (AR200; Reichert).

### ClearSee protocol

ClearSee solutions were prepared by mixing xylitol powder [#04; final 10% (w/v)], sodium deoxycholate [#07; final 15% (w/v)] and urea [#19; final 25% (w/v)] in water. Seedlings, leaves and pistils of *A. thaliana* and gametophores of *P. patens* were fixed with 4% (w/v) PFA for 30-120 min (seedlings, 30 min; leaves, 120 min; pistil or gametophores, 60 min) in PBS under vacuum (∼690 mmHg) at room temperature. Fixed tissues were washed twice for 1 min each in PBS and cleared with ClearSee at room temperature for 4 days to 4 weeks or more, depending on tissue type. The minimum incubation times for clearing were 4 days for leaves, roots and moss, 7 days for seedlings, 2 weeks for pistils, and 4 weeks for mature stems. In the case of pistils, incubation for 4 weeks improved clarity. ClearSee-treated samples could be stored at room temperature for at least 5 months. For post-staining, cleared tissues were stained with Calcofluor White (final 100 µg/ml) in ClearSee solution for 1 h, and Hoechst 33342 (final 10 µg/ml) in ClearSee solution overnight. After staining, tissues were washed in ClearSee for 1 h.

### Microscopy settings

For screening of chemical reagents and deep imaging, we used three microscope systems. Settings are detailed in the supplementary Materials and Methods.
